# Sense of personal control: Can it be assessed culturally unbiased across Aboriginal and non-Aboriginal Australians?

**DOI:** 10.1371/journal.pone.0239384

**Published:** 2020-10-01

**Authors:** Pedro Henrique Ribeiro Santiago, Tine Nielsen, Rachel Roberts, Lisa Gaye Smithers, Lisa Jamieson

**Affiliations:** 1 Australian Research Centre for Population Oral Health (ARCPOH), Adelaide Dental School, The University of Adelaide, Adelaide, South Australia, Australia; 2 Department of Psychology, The University of Copenhagen, Copenhagen, Denmark; 3 School of Psychology, The University of Adelaide, Adelaide, South Australia, Australia; 4 School of Public Health, The University of Adelaide, Adelaide, South Australia, Australia; University of Copenhagen, DENMARK

## Abstract

In recent decades, several studies have emphasized sense of personal control as a prominent aspect of Aboriginal health. However, one limitation is that instruments available to measure personal control were originally developed in western countries and validation for Aboriginal Australians has not been conducted. The aims of the current study were to evaluate whether the Sense of Personal Control Scale (SPCS) can be used to obtain culturally unbiased measurement of personal control across Aboriginal and non-Aboriginal Australians and to assess the psychometric properties of the SPCS for Aboriginal and non-Aboriginal Australian. Methods: The current study utilized two Australian subsamples retrieved from the Teeth Talk Study (n = 317) and the National Survey of Adult Oral Health 2004–2006 (n = 3,857) in which the SPCS was included. Graphical Loglinear Rasch Models (GLLRM) were used to fulfill the aims of the study. Results: The Perceived Constraints subscale fitted a GLLRM for Aboriginal Australians after the exclusion of three items, while fit to any Rasch model (RM) or GLLRM model could not be found in the non-Aboriginal sample. The Mastery subscale fitted a GLLRM in the non-Aboriginal sample after the exclusion of one item. In the Aboriginal sample, two items of the Mastery subscale fitted the RM, however, two items cannot be considered as a scale. Conclusion: In the present study, we showed that the development of new items is crucial before the revised SPCS might constitute a valid and reliable measure of sense of personal control in both Aboriginal and non-Aboriginal Australian populations, and it is possible to assess whether the SPCS can be measured without bias across these two populations.

## Introduction

A topic of on-going research in Australia is how social determinants of health contribute to the large health inequalities between Aboriginal and non-Aboriginal Australians [1]. Among different social determinants, recent studies have emphasized sense of personal control as a fundamental aspect of the contemporary Aboriginal experience [2]. Sense of personal control is the generalized expectation that outcomes are contingent on individual behaviours [3]. Therefore, it has operationalized into two dimensions: *perceived constraints*, beliefs that outcomes are beyond individual influence, being determined by external factors [4]; and *mastery*, beliefs that individual behaviours will produce the desired outcomes [5].

The effects of personal control on health have been extensively studied in non-Indigenous populations. Meta-analyses have associated sense of personal control with general well-being (mental well-being, life satisfaction and physical health), higher job satisfaction [6], lower burnout [7] and lower depression [8]. Individual studies have also associated personal control with anxiety [9] and longevity [10].

### Sense of personal control of Aboriginal Australians

In Australia, the legacy of colonization and subsequent decades of assimilation policies had a direct impact on the sense of personal control of Aboriginal people. Aboriginal Australians were marginalized from participation in major social and political decisions and their society was disassembled during the 20^th^ century. The undermining of self-determination in social matters, both in the country and in their communities, led individuals to lose the sense of control over their lives [11].

A few recent studies have investigated the effects of personal control on Aboriginal health. Daniel, Brown [11] examined personal control in a remote Aboriginal community with poor living conditions (e.g. limited access to transportation, communication, food storage) and found it to be negatively associated with stress. Furthermore, considering that racism against Aboriginal people creates unfair and unpredictable demands that can undermine personal control [12], a recent study by Paradies and Cunningham [13] showed that personal control mediated the effects of racism on depression. Finally, Reilly, Doyle [2] suggested personal control as a potential protective factor of cardiovascular disease and recommended “further empirical investigation” in Aboriginal populations.

One instrument created to evaluate sense of personal control in non-Indigenous populations is the Sense of Personal Control Scale (SCPS). The SPCS was originally developed based on a widely used instrument to measure personal control: the seven-item Pearlin Mastery Scale [4], which was later expanded with new five items to create the SPCS (S1 Table). The validity of Pearlin’s 7-item Mastery Scale has been investigated in several cultures, for example in countries such as Sweden, Iran, China, Japan, among others [14–16]. Moreover, the 7-item Mastery Scale scale has also been examined with modern psychometric methods, including Rasch models [15] and translated to Indigenous languages such as the Yolngu Matha, an Aboriginal language spoken in northeast Australia [11]. Despite the investigation of the 7-item Mastery Scale psychometric properties in several countries, no previous study conducted a cross-cultural validation.

On the other hand, the psychometric properties of the extended 12-item SPCS have not been evaluated in other cultures since the original study, which employed an American mainly Caucasian sample [17]. Prior to application with Aboriginal participants in the original data collection, we followed recommendations for the cultural adaptation of psychological instruments [18] and consulted an Aboriginal reference group with 15 members, comprising Aboriginal community members and Aboriginal Infant Care workers, to ensure the appropriateness of the instrument in the Aboriginal population [19]. The reference group examined the items of the SPCS and indicated that the instrument had content and face validity for Aboriginal cultures. Thus, as face and content validity are requirements of more encompassing forms of validity such as construct validity, we found the SPCS to be suitable for further psychometric analysis in Aboriginal Australians.

## Unbiased measurement of personal control of Aboriginal and non-Aboriginal Australians

While research on the effects of personal control on Aboriginal health is on-going, there are two main gaps that this study aims to address. Firstly, it is important to investigate whether psychological instruments can provide unbiased measurement of personal control for Aboriginal Australians compared with non-Aboriginal Australians. The importance of cross-cultural comparison is that the validation of an instrument for Aboriginal Australians can only inform the level of the construct measured (e.g. level of personal control) *within* the Aboriginal community (and likewise for non-Aboriginal Australians). It is the development of culturally unbiased instruments that can inform the real impact of social inequalities on Aboriginal Australians’ sense of personal control by *comparing it* to a non-Aboriginal group. That is, a culturally unbiased instrument can inform how much personal control Aboriginal Australians experience by contrasting their personal control with that of non-Aboriginal Australians.

Secondly, recent recommendations by Santiago and colleagues [20] emphasized the importance of validating psychological instruments specifically for Aboriginal Australians and it seems particularly important to validate measures of personal control for culturally defined subpopulations since personal control is influenced by culture [21]. For example, the association of personal control with anxiety symptoms is weaker in collectivist societies compared to individualistic (western) societies [21]; moreover, individuals from collectivist cultures (e.g. China) are more likely to exert control through cultivating relationships, while individuals from individualistic cultures tend to exert control through personal effort (e.g. US) [22]. Since Aboriginal Australians comprise several collectivist cultures [23] and the general (non-Aboriginal) Australian population form a western individualistic society (in most part due to its European descendants), these cultural differences raise questions whether western-developed measures of personal control are appropriate for Aboriginal Australians. Previous studies applied sense of personal control measures without validation for Aboriginal people [11]. It is necessary to ensure that psychological instruments have construct validity *specifically* for Aboriginal and Torres Strait Islanders in Australia, otherwise the item responses can lead to biased scores.

### The present research

The first and main aim of the study was to investigate whether the SPCS can be used to obtain culturally unbiased measurement of personal control across Aboriginal and non-Aboriginal Australians. To do this, we first needed to evaluate the construct validity and the psychometric properties of the SPCS separately for Aboriginal Australians and non-Aboriginal Australians (i.e. does the SPCS measure the proposed constructs for both groups), which comprises the secondary aim of this study. To achieve these two aims, we employed state-of-the-art item response theory methods in the form of Rasch models and graphical loglinear Rasch models to conduct detailed item analysis of the SPCS within and across samples of Aboriginal and non-Aboriginal Australians.

## Methods

### Measures

Sense of Personal Control Scale (SPCS): The SPCS is a 12 item scale intended to measure sense of personal control [17] consisting of two subscales, Perceived Constraints (PC) and Mastery (MA). Items were rated on a five-point response scale (1 = Not at all, 2 = Rarely, 3 = Sometimes, 4 = Fairly often, 5 = Very often) (S1 Table). Since the SPCS comprises two subscales, total scores should be calculated for each subscale independently. Total scores for the MA subscale range from 4 to 20, with higher scores indicating higher mastery. Total scores for the PC subscale range from 8 to 40, with higher scores indicating higher perceived control. In the original development and validation study of the SPCS, Exploratory Factor Analysis (EFA) found a two-dimensional structure, interpreted as Perceived Constraints and Mastery. Internal consistency of the PC subscale was α = .86 and for the MA subscale α = .70 [17].

### Exogenous variables

The exogenous variables of sex, age, education and employment were chosen to be included in this study for the evaluation of differential item functioning. The reason for their inclusion is that there is strong empirical evidence that sense of personal control differs according to the aforementioned characteristics. For instance, Ross and Mirowsky [24] reported that men display higher sense of personal control than women and this difference was larger in older compared to younger groups. Among the reasons why women, on average, display lower sense of personal control than men includes unfairness in the division of household labour, a gender pay gap and more restricted opportunities for certain jobs. On the other hand, education increases levels of personal control. People with higher education frequently meet other people who are attentive, career-driven and persistent, providing them opportunities to learn confidence and self-assurance. Education also serves as an avenue for socioeconomic status and, consequently, personal control over the circumstances [24].

Considering that exogenous variables such as sex, age, education and employment were shown by empirical research to be main influences on personal control, it is necessary that instruments correctly measure personal control in these groups (e.g. men/women), so group differences reflect true personal control differences. For this reason, we included these characteristics in our analysis. Education was measured through the categories “Up to High school” and “Technical/Tertiary education”. Since items measuring education had a different number of categories in each sample (i.e. Aboriginal and non-Aboriginal Australians), we dichotomized the variable into two categories to enable the comparison across samples. Employment status was measured through the categories “Employed” and “Unemployed”. Once again, we dichotomized this variable to enable comparison across samples. Finally, age was dichotomized into groups aged up to 45 years old and more than 45 years old.

### Samples

The current study utilized two Australian subsamples retrieved from other studies, where the SPCS had been included in the collected data. The first sample was composed of 317 Aboriginal Australians that participated in the Teeth Talk study [19]. The Teeth Talk study was a randomized controlled trial (RCT) aimed at improving oral health literacy among Aboriginal adults in South Australia. The study was promoted via posters in community centres and advertisements on a local radio station. The participants were recruited through various methods, including home visits, referrals, self-nomination and word of mouth.

The second sample was composed of 3,857 non-Aboriginal Australians in the population-based cross-sectional study Australia’s National Survey of Adult Oral Health (NSAOH) 2004–2006 [25]. The study used a questionnaire that was mailed to participants that undertook dental examination. The National Study of Adult Oral Health (NSAOH) 2004–2006 was approved by the University of Adelaide's Human Research Ethics Committee. The Teeth Talk (TT) study received ethical approval from the Aboriginal Health Council of South Australia, the Human Research Ethics Committee of the University of Adelaide, the Board ofManagement of the Pika Wiya Health Service (PWHS) and the local community controlled Indigenous health service. All participants provided signed informed consent. The demographic characteristics of each sample are included in S2 Table.

### Rasch measurement models

The class of Rasch models belongs to the larger family of item response theory (IRT) models. The simplest is the original Rasch Model (RM) for dichotomous items [26]. In the current study, we used the Partial Credit Model (PCM) [27], which generalize the RM to ordinal items, and Graphical Log-Linear Rasch models (GLLRM), which can take both dichotomous and ordinal items [28–30]. As both the dichotomous RM and the ordinal PCM adhere to the same requirements for measurement [31, 32], we use the term “RM” for Rasch model for both in the remainder of the paper. The five basic requirements for measurement are: 1) *unidimensionality*, the items of a scale or subscale measure a single underlying latent construct—e.g. personal control; 2) *monotonicity*, the expected item scores increase with increasing values on the latent variable. Thus, increasing levels of personal control increases the probability of endorsing item categories that indicate more personal control—e.g. “very often” instead of “rarely”; 3) *local independence of items* (or no LD), the item responses are conditionally independent given the latent variable. Thus responses to an item depend only on the level of personal control, and not systematically on responses to any of the other items; 4) *absence of differential item functioning* (no DIF), item responses and exogenous variables (i.e. relevant background variables) are conditionally independent given the latent variable. Thus, the responses to an item depend only on the level of personal control, and not systematically on subgroup membership such as culturally defined groups; and 5) *homogeneity*, the rank order of item parameters (item “difficulties”) is the same for all persons no matter their level on the latent variable. Thus, the items which require the relatively lowest and highest levels of personal control to be endorsed are the same for persons with all levels of personal control.

The first four requirements above provide criterion-related construct validity according to Rosenbaum [33] and are common for all IRT models. The fifth requirement of homogeneity pertains only to the RM. Fulfilment of all five requirements provides ideal measurement, as the raw summed score is then a sufficient statistic for the estimated person parameter. Sufficiency of the raw sum score distinguishes scales fitting Rasch models from scales fitting other IRT models [31]. Sufficiency is desirable when summed raw scores are used, such as is the case with sense of control in population surveys [34]. However, it is also possible to convert the sum scores to Rasch scores (i.e. the estimated person parameters), which are on a logit scale, if preferred.

It is common that quality-of-life related scales do not fulfil all five requirements of the RM, and thus not fit the RM. In such cases, it is still possible to achieve close to optimal measurement, if the only departures from the model are in the form of uniform DIF and/or uniform LD [28, 30, 34]. Uniform implies that the LD or DIF is the same across all levels of the latent construct. Uniform LD and DIF can be adjusted for in GLLRMs, which are extensions of the RM that model these two specific departures from the RM. When a GLLRM adjusts only for uniform LD, the sufficiency of the sum score is not affected, but the reliability of the instrument often appear lower than when LD is not taken into account. If a GLLRM includes uniform DIF, the sum score is no longer a sufficient statistic for the person parameter; however, adjusting the sum scores for DIF enables subsequent comparisons of subgroup scores without measurement bias [34]. Similarly, person parameters can be estimated in each subgroup without bias. GLLRMs might thus provide a solution to overcome cultural bias between the personal control scores of Aboriginal and non-Aboriginal Australians. The cultural bias can be detected in the item analysis and, after correction, facilitate unconfounded comparison of sense of personal control between these two groups.

### Item analysis by Rasch and graphical loglinear Rasch models

#### Statistics

Overall tests of fit to RMs or GLLRMs (i.e. tests of global homogeneity by comparison of item parameters in low and high scoring groups, and tests of no DIF) were conducted using Andersen [35] conditional likelihood ratio test (CLR). The fit of individual items was tested by comparing the observed item-rest-score correlations with the expected item-restscore correlations under the model [30] and with conditional infit and outfit statistics [36]. The lack of local independence and DIF was tested in two ways: (a) conditional tests of independence using partial Goodman-Kruskal gamma coefficients for the conditional association between item pairs (indicating the presence of LD) or between items and exogenous variables (indicating the presence of DIF) given the restscores [30]; and (b) Kelderman’s [37] conditional likelihood ratio test of no DIF/no LD. Evidence of overall homogeneity and no global DIF found in the global tests was rejected if this was not supported by individual item fit and absence of LD and/or DIF at the item level.

The Benjamini-Hochberg procedure was used to adjust for false discovery rate (FDR) due to multiple testing [38], in order to reduce false evidence against the model created by the many tests conducted (i.e. reduce type I errors).

All the test statistics described above effectively test whether data comply with the expectations of the model in question, and thus the results are all evaluated in the same manner: significant p-values indicate evidence against the model. In line with the recommendations by Cox, Spjøtvoll [39], we did not apply a critical limit of 5% for p-values as a deterministic decision criterion, but we used p-values as a continuous measure of evidence against the null, distinguishing between weak (p < 0.05), moderate (p < 0.01), and strong (p < 0.001) evidence against the model.

Reliability was estimated using Hamon and Mesbah [40] Monte Carlo method, which takes into account any local dependence between items in a GLLRM and adjusts the reliability accordingly (in contrast to Cronbach’s α, which require local independence of items). Targeting was assessed numerically by the Test Information (TI) target index, which is the mean test information divided by the maximum test information [36], with a value of one indicating perfect targeting. For a graphical illustration of targeting and test information, we plotted item maps showing the distribution of the item thresholds against weighted maximum likelihood estimates of the person parameters and the person parameter estimates assuming a normal distribution (i.e. the theoretical distribution), and included the information function. All item analysis was conducted with Digram software [41, 42] and item maps were created with R software [43].

#### Strategy of analysis

The SPCS was developed to include two subscales, Perceived Constraints and Mastery. However, as the development study [17] employed only exploratory methods to determine these two subscales, we found it appropriate to conduct an initial item analysis of the full 12-item SPCS to confirm that our strategy to analyse the subscales separately was correct. Considering the cultural differences between the two subsamples, our approach was to first conduct the item analysis independently in each of the Aboriginal and non-Aboriginal Australians samples and secondly to analyse the samples jointly. With this approach, it would be easier to determine any DIF related to cultural differences in the analysis of the combined sample, as any other measurement issue with items had already been discovered in each of the samples.

The item analysis of both the full SPCS scale and each of the PC and MA subscale in each sample followed the same overall strategy. Initially, fit to the RM was assessed with the global tests of homogeneity and DIF, fit of individual items, as well as analysis of local independence and DIF at the item level. If a scale did not fit the RM, we proceeded to catalogue the departures, and if these consisted only of uniform LD or DIF, we proceeded to test the fit of the item responses to a GLLRM. When we were not able to successfully define a GLLRM, we eliminated the most (statistically and content-wise) problematic item and proceeded again to test fit to the RM for the reduced scale and so on, in an iterative process. Thus, the analyses of each scale in each sample consisted of numerous iterations, and each iteration consisted of several statistical tests and steps.

## Results

The demographic characteristics of both samples are found in S2 Table. The Aboriginal sample had an average age of 36.4 (SD = 14.0, range = 18–82), 76% of participants were women, 26% had tertiary education and 25% were employed. The non-Aboriginal Australian sample had an average age of 50.3 (SD = 14.8, range = 18–82), 62% of participants were women, 67% had tertiary education and 76% were employed. Hence, compared to the non-Aboriginal Australians, the Aboriginal participants were largely socially-economically disadvantaged.

### Preliminary analysis of the full 12-item Sense of Personal Control Scale

In this section, we briefly describe the results of the preliminary analyses of the full 12-item SPCS.

In the Aboriginal Australians sample, the 12-item SPCS scale did not fit the RM (S3 Table). We proceeded by investigating whether departures in terms of LD/DIF could be adjusted by GLLRM and by removing misfitting items one by one throughout the iterations. Items 11, 4, 1 and 3 were sequentially excluded, thus the entire MA subscale. Therefore, it became clear that the items from the MA subscale did not measure the same construct as the items from the PC subscale and both subscales could not form a unidimensional model in the Aboriginal sample. Similarly, the 12-item SPCS did not fit the RM for the non-Aboriginal Australian sample (S3 Table). The item analysis again indicated that the items from the MA subscale did not measure the same construct as the items from the PC subscale.

Based on these results, we were confident that our chosen strategy of analysing the PC and the MA subscales separately was correct, and we thus proceeded accordingly.

### Item analysis of the Perceived Constraints and Mastery subscales

#### Perceived Constraints subscale in the Aboriginal sample

The 8-item PC subscale did not fit the Rasch Model (RM) at overall (S4 Table, see PC Aboriginal Australians column) or the item level (S5 Table, see PC Aboriginal Australians rows). We proceeded to investigate whether the departures consisted of LD and DIF and could be adjusted with GLLRM. However, we were unable to fit a GLLRM for the complete PC subscale with all 8 items. After several iterations investigating model departures, the misfitting items 6 (“I often feel helpless in dealing with life’s problems”), 8 (“I have little control over the things that happen to me”) and 9 (“There is really no way I can solve all the problems I have”) were excluded, and overall fit to a GLLRM with LD among all items pairs and no DIF according to sex, age, education and employment status was established (Table 1, see PC Aboriginal Australians column).

**Table 1:** Overall fit statistics for the resulting Rasch and graphical loglinear Rasch model for the PC and MA subscales^§^.

Overall Tests	PC (Aboriginal Australians)^a^			MA (Aboriginal Australians)^b^			MA (non-Aboriginal Australians)^c^		
	CLR	*Df*	*p*	CLR	*df*	*p*	CLR	*df*	*p*
Homogeneity	89.6	80	.22	12.5	6	.05	52.6	34	.02
Global DIF relative to:									
Sex	92.1	80	.17	5.4	6	.50	29.3	26	.30
Age	96.8	80	.10	6.9	6	.33	38.2	30	.14
Education	115.9	80	.02	8.2	6	.22	42.4	34	.15
Employment status	107.9	80	.005	12.0	6	.06	56.6	34	0.009

*Notes*. PC: Personal Constraints Scale. MA: Mastery Scale. CLR: Conditional likelihood ratio. df: degrees of freedom. p: p-value. DIF: differential item function. Overall homogeneity test compares item parameters in approximately equal-sized groups of high and low scoring persons, while the global DIF test for DIF across the entire set of items. The critical limits for the p-values after adjusting for false discovery rate in the GLLRM were: (a)(b) 5% limit p = .01 and 1% limit p = .002; (c) 5% = 0.05 and 1% limit p = .002.

^§^The results displayed in this table refer to the reduced subscales after the exclusion of misfitting items.

**Fig 1:**
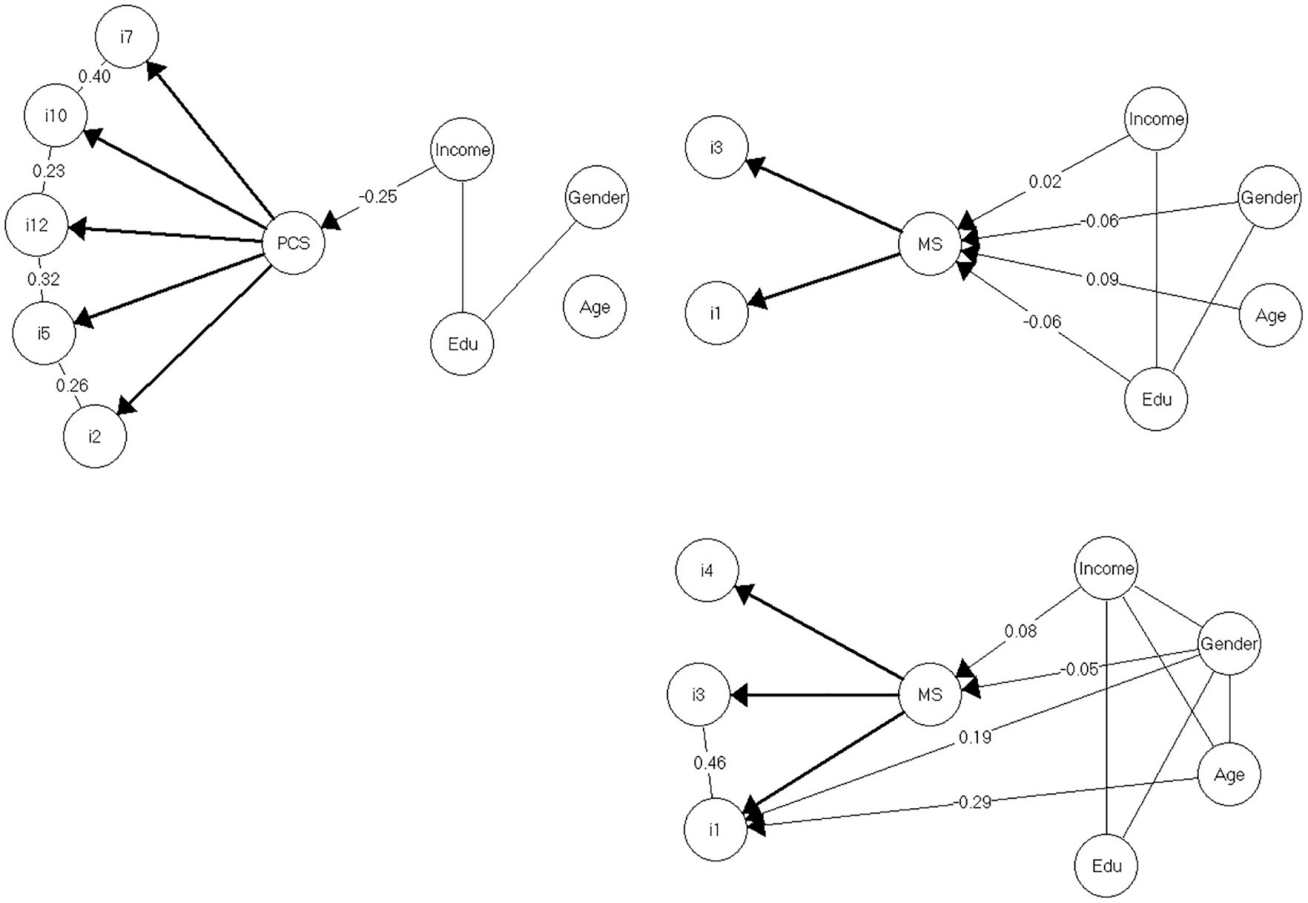
Resulting models for the Perceived Constraints and Mastery subscales. *Note*. Graphical loglinear Rasch model for the Perceived Constraints subscale for Aboriginal Australians (top left), Rasch model for the Mastery subscale for Aboriginal Australians (top right) Graphical loglinear Rasch model for the Mastery subscale for non-Aboriginal Australians (bottom right). Disconnected nodes indicate that variables are conditionally independent and partial gamma coefficients (Goodman & Kruskal’s *γ*) informs the magnitude of the LD and DIF.

Moreover, there were no issues with item fit for the five retained items of the PC subscale (Table 2, see PC GLLRM Aboriginal Australians rows). The GLLRM is displayed in Fig 1.

**Table 2:** Item fit statistics for the final GLLRMs and RM of the PC and MA subscales.

	Conditional Infit			Conditional Outfit			Item-restscore association		
Items^§^	Observed	SE	*p*	Observed	SE	*p*	Observed ^γ^	Expected ^γ^	*p*
PC GLLRM									
*Aboriginal Australians*									
2	0.958	0.080	0.600	0.936	0.080	0.427	0.384	0.351	0.517
5	1.033	0.088	0.705	1.039	0.091	0.671	0.448	0.456	0.874
7	0.998	0.081	0.981	0.989	0.079	0.892	0.409	0.384	0.622
10	1.031	0.084	0.709	1.085	0.090	0.349	0.463	0.451	0.799
12	1.004	0.088	0.965	0.991	0.098	0.924	0.469	0.470	0.983
MA RM									
*Aboriginal Australians*									
1	1.091	0.112	0.418	0.997	0.145	0.991	0.809	0.799	0.784
3	1.091	0.112	0.418	0.997	0.145	0.991	0.809	0.799	0.784
MA GLLRM									
*non-Aboriginal Australians*									
1	1.073	0.032	0.02	1.004	0.048	0.94	0.701	0.711	0.490
3	0.936	0.038	0.08	0.859	0.057	0.01	0.786	0.754	0.021
4	1.011	0.025	0.66	0.962	0.043	0.38	0.596	0.579	0.334

*Notes*.

^γ^ = Goodman & Kruskal’s gamma coefficients.

PC: Perceived Constraints Scale. MA: Mastery Scale. RM: Rasch model. GLLRM Graphical loglinear Rasch model.

^§^The results displayed in this table refer to the reduced subscales after the exclusion of misfitting items.

Regarding the LD found between all items pairs, content evaluation confirmed a large conceptual overlap between the PC items. For example, item 10 states that “I sometimes feel as I am being pushed around in my life” while item 7 states that “There are many things that interfere with that I want to do”. It seems reasonable that if a person has been “pushed around in life”, there will be “many things” interfering with what that person wants to do. Thus, this seems to be a case of positive *response dependence*, in which endorsement of one item logically imply the endorsement of another [44]. Response dependence is also probably involved in the local dependence found between the other PC items.

One important reason of accounting for LD is that, if a reliability index that assumes local independence such as the Cronbach’s [45] alpha was applied to the 5 items of the revised PC subscale, the result would have indicated adequate reliability (α = 0.71–95% CI [0.66, 0.76]). However, after calculating reliability by adjusting for LD among all items pairs through Hamon and Mesbah [42] Monte Carlo method, the results indicated that the *true* reliability was poor (R = 0.54).

**Fig 2:**
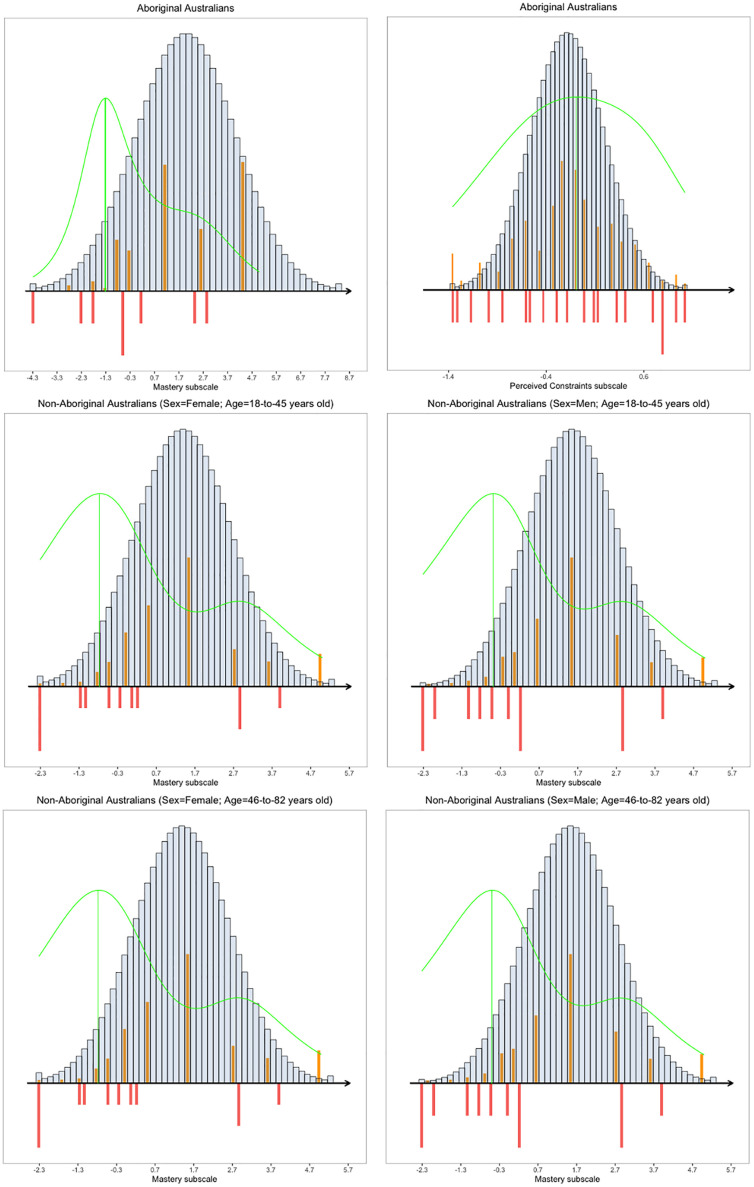
Item map of the PC subscale and MA subscale for Aboriginal and non-Aboriginal Australians. *Note*. The orange bars display the person parameters (WML estimates). The grey bars display the population distribution under the assumption of normality. The red bars display the item thresholds and the green line is the Fisher’s information function.

Finally, the targeting of the PC subscale for Aboriginal Australians was excellent. For example, the TI target index indicated that for the Aboriginal sample the PC subscale provided 94% of the total information available if the scale was perfectly targeted. The PC subscale ranged from “easy” items (Item 10 –“I *sometimes* feel I am being pushed around in my life”), which were endorsed by participants with low perceived constraints, to “difficult” items (Item 7 –“There are *many* things that interfere with what I want to do”), which were endorsed by those with high perceived constraints. The item maps (Fig 2) showed that the 5 items covered the whole range of perceived constraints in the Aboriginal population.

#### Mastery subscale in the Aboriginal sample

The full 4-item MA subscale did not fit the Rasch model either on overall (S4 Table, see MA Aboriginal Australians column) or item level (S5 Table, MA Aboriginal Australians rows). Since the model departures did not consist uniquely of LD and DIF, departures could not be adjusted for in a GLLRM. After the exclusion of two misfitting items, item 4 (“Whether or not I am able to get what I want was in my own hands”) and item 11 (“What happens to me in the future mostly depends on me”), the two remaining items, item 1 (“I can do just about anything I really set my mind to”) and item 3 (“When I really want to do something I usually find a way to”), fitted the RM at overall (Table 1, see MA Aboriginal Australians column) and item level (Table 2, see MA RM Aboriginal Australians rows). However, only two items cannot be considered as a scale, and so be interpreted with caution. The targeting of the 2-item MA subscale for Aboriginal Australians was poor, since the TI target index ranged from .42 to .56 across the subgroups showing DIF, and reliability was adequate (R = 0.75).

#### Perceived Constraints subscale in the non-Aboriginal sample

The 8-item PC subscale did not fit the RM at an overall (S3 Table) or item level (S4 Table). Despite several iterations investigating model departures, it was not possible to fit any model (RM or GLLRM) for the PC subscale in the non-Aboriginal sample. For this reason, reliability and targeting could not be calculated.

#### Mastery subscale in the non-Aboriginal sample

The 4-item MA subscale did not fit the RM (S3 and S4 Tables), and a GLLRM adjusting for LD and/or DIF could not be established either. After the exclusion of item 4 (“Whether or not I am able to get what I want is in my own hands”), the remaining 3 items fitted a GLLRM with age and gender DIF for item 1, as well as LD between items 1 and 3 (Tables 1 and 2). The targeting of the MA subscale for non-Aboriginal Australians was adequate, since the TI target index ranged from .72 to .75 across the DIF-defined subgroups, and reliability was poor (R = 0.64).

### Cross-cultural comparison

Considering that only two items, Item 1 and 3 of the MA subscale, functioned for both Aboriginal and non-Aboriginal Australians, we were not able to proceed with the cross-cultural analyses of any of the two SPCS subscales, including testing for DIF across both cultures. Therefore, the SPCS did not provide unbiased measurement across Aboriginal and non-Aboriginal Australians and we were not able to achieve the main aim of this study.

## Discussion

The main aim of the present study was to evaluate whether the SPCS could be used to obtain culturally unbiased measurement of personal control across Aboriginal and non-Aboriginal Australians, and thus also to investigate the psychometric properties of the SPCS in an Aboriginal and a non-Aboriginal Australian population. The findings indicated that: (a) the SPCS did not provide culturally unbiased measurement across Aboriginal and non-Aboriginal samples; (b) a revised 5-item PC subscale was a measure of perceived constraints for Aboriginal Australians, however, the overlap in content among items led to poor reliability; and (c) the revised MA subscale had only 2 items so new culturally-specific items should be developed before its application in Aboriginal Australians.

### Unbiased measurement across Aboriginal and non-Aboriginal Australians

In the current study, we were not able to conduct cross-cultural comparisons across Aboriginal and non-Aboriginal Australians. This finding reinforces that comparability of scores from psychological instruments between Aboriginal and non-Aboriginal groups should not be assumed and measurement invariance across these cultures needs to be investigated prior to the use of test scores in cross-cultural research. Regarding sense of personal control, we recommended that future studies should modify and extend the SPCS scales based on the current results (e.g. using Nielsen & Kreiner’s [46] strategy for item improvement). After implementing improvements, the validity and reliability of these new scales should be again investigated, before assessing whether they are suitable for cross-cultural and unbiased measurement across Aboriginal and non-Aboriginal Australians.

### Psychometric properties of the SPCS for Aboriginal Australians

The findings indicated that the SPCS is composed of two subscales, PC and MA. Thus, in the future use of the SPCS with Aboriginal Australians, total scores need to be computed for the PC and MA subscales independently (instead of summing across all items).

#### Perceived Constraints subscale

One main finding was that all five items of the revised PC were locally dependent due to the large conceptual overlap between these items. One practical consequence of local dependence is diminished reliability and inflated estimates of reliability when methods that do not adjust for LD, such as Cronbach’s α, are calculated. The reason for the diminished reliability is that since items are too conceptually similar, they are not different enough to provide one item worth of information [47]. Thus, although the revised 5-item PC subscale provides a potentially valid measure of perceived constraints among Aboriginal Australians, measurement was not reliable in this sample for research purposes and even less for individual assessment [48]. This result is worrisome since, although Aboriginal Australians comprise several culturally distinct groups, this population is notably homogeneous due to their experience of social inequalities as a whole [1]. Hence, when psychological assessment is performed with Aboriginal Australians, the low trait variance (respondents are similar) needs to be compensated with higher measurement precision [20]. These findings imply that future studies need to develop culturally sensitive items to improve the PC subscale.

The 5 PC items displayed no DIF by sex, age, employment status or education. Therefore, scores (and person parameters) can be compared across these groups and will reflect true differences in perceived constraints rather than measurement bias. Nonetheless, the Aboriginal sample had a moderate size and future studies should investigate DIF in larger samples (i.e. more statistical power) to confirm no DIF by these items.

#### Mastery subscale

The analysis indicated that 2 MA items fit the RM. However, although in this case the items were locally independent, two items cannot be considered as a scale and the results need to be interpreted with caution. The development of the SPCS by Lachman and Weaver [17] included item 3 (“When I really want to do something I usually find a way to do it”) and item 4 (“Whether or not I am able to get what I want is in my own hands”), which were added to the original items 1 (“I can do just about anything I really set my mind to”) and 11 (“What happens to me in the future mostly depends on me”) present in the Pearlin [4] Mastery Scale. These included items, item 4 and 11, were the ones which displayed misfit and were excluded. Problems with item 11 have been previously reported. For example, when evaluating the Pearlin [4] Mastery Scale with Rasch analysis, Eklund, Erlandsson [15] showed that item 11 had the most pronounced misfit among the items and that this item “may represent a different construct than the one measured by the scale as a whole”. Therefore, in agreement with Eklund, Erlandsson [15], we recommend item 11 to be excluded.

After the exclusion, the two remaining items do not constitute a scale and it is implausible that they would cover enough content of a multifaceted construct such as mastery [49], posing immediate concerns of construct underrepresentation [50]. For this reason, future studies should include culturally-specific items to evaluate mastery in Aboriginal Australians. Among these new items, one recommendation is the inclusion of items to measure *communal mastery* rather than *personal mastery*. While personal mastery promotes coping through individualized strategies, communal mastery improves coping through the use of the social network [51]. In a study with Indigenous American women, Hobfoll, Jackson [52] showed that, while personal mastery was a strong predictor for coping with stress in individualistic cultures, communal mastery is more effective in enhancing coping in collectivistic cultures such as Indigenous populations. Due to these considerations, psychological instruments that measure both personal and communal mastery have been developed and one was recently validated in a *Yup’ik* population, an Indigenous group of Alaska natives [50]. One example of an item measuring communal mastery is “What happens to me in the future depends on my ability to work well with others” [53], contrasting directly with item 11 (“What happens to me in the future mostly depends on me”) which was eliminated due to misfit in the current study.

### Psychometric properties of the SPCS for non-Aboriginal Australians

The psychometric properties of the SPCS were poor for non-Aboriginal Australians. It was not possible to obtain fit to a model with the Perceived Constraints subscale and the 3-item Mastery subscale had several problems in terms of DIF and LD. Once again, in the MA subscale, the most problematic item was Item 11 (“What happens to me in the future mostly depends on me”) which was excluded. The fact that this item was removed in both cultures (Aboriginal and non-Aboriginal), alongside with previous studies such as Eklund, Erlandsson [15], provides further evidence that Item 11 is possibly measuring a distinct construct. We hypothesize that the problem is with the item wording since the statement “what happens to me in the future mostly depends on me” can plausibly be rejected by respondents with low mastery *and* by respondents with high mastery. That is, even participants with high mastery, who believe their individual behaviours will produce desired outcomes, can possibly acknowledge that the future is *mostly* unpredictable and does not depend on them. Despite the SPCS being originally developed in a Western country [17], both subscales did not work adequately for non-Aboriginal Australians, indicating problems with the instrument.

### Strengths and limitations

The strengths of the present study include the use of item response theory methods to evaluate issues of DIF and LD. Another strength is the size of the sample used for validation of psychological instruments for Aboriginal Australians; due to notably difficulty in recruiting participants from Indigenous populations, this is one of the best datasets available for investigating the psychometric properties of a sense of personal control measure in an Aboriginal population. Moreover, we also employed a large non-Aboriginal sample for the analysis of cross-cultural validity. Limitations include the fact that the Aboriginal sample was a convenience sample in a rural setting and was composed mostly of women. Therefore, it is unclear whether the analysis had enough power to detect DIF by sex, and the absence of DIF by sex needs to be replicated in independent Aboriginal samples. Furthermore, many exogenous variables present in the original studies were not comparable across Aboriginal and non-Aboriginal Australians, which limited our possibilities of analysis of cross-cultural validity, and thus the issue of culturally unbiased measurement across the two cultures.

## Conclusions

In the present study, we showed that the development of new culturally-specific items is needed before the revised SPCS might constitute a valid and reliable measure of sense of personal control in both Aboriginal and non-Aboriginal Australian populations, thus making it possible to assess whether the SPCS can provide culturally unbiased measurement across these two populations.

## Supporting information

S1 Table The Sense of Personal Constrol Scale (SPCS) items compared to items in Pearlin’s Mastery Scale. (DOCX)

S2 Table Characteristic of the study participants. Mean values, minimum, maximum and standard deviations; numbers and percentages. TAFE, Technical and Further Education (trade school/college).
(DOCX)

S3 Table Overall fit statistics for the Rasch model for the 12-item SPCS. CLR: Conditional likelihood ratio. df: degrees of freedom. p: p-value. DIF: differential item function. Overall homogeneity test compares item parameters in approximately equal-sized groups of high and low scoring persons, while the global DIF test for DIF across the entire set of items. The critical limits for the p-values after adjusting for false discovery rate in the GLLRM were: (a)(b) 5% limit p = .05 and 1% limit p = .01.
(DOCX)

S4 Table Overall tests of fit to the Rasch model for the PC and MA subscales. PC: Personal Constraints Scale. MA: Mastery Scale. CLR: Conditional likelihood ratio. df: degrees of freedom. p: p-value. DIF: differential item function. Overall homogeneity compares item parameters in approximately equal-sized groups of high and low scoring persons, while the global DIF test for DIF across the entire set of items. The critical limits for the p-values after adjusting for false discovery rate in the GLLRM were: (a) (b) 5% limit p = .05 and 1% limit p = .01; and (c) (d) 5% limit p = .05 and 1% limit p = .01. § The results displayed in this table refer to the original subscales with all items included.
(DOCX)

S5 Table Item fit statistics for the Rasch model of the PC and MA subscales for Aboriginal Australians. γ = Goodman & Kruskal’s gamma coefficients. PC: Perceived Constraints Scale. MA: Mastery Scale. § The results displayed in this table refer to the original subscales with all items. The critical limits for the p-values after adjusting for false discovery rate in the GLLRM were: (a) 5% limit p = .02 and 1% limit p = .003; (b) 5% limit p = 0.02 and 1% limit p = .004; (c) 5% limit p = 0.04 and 1% limit p = .008; and (d) 5% limit p = 0.04 and 1% limit p = .007.
(DOCX)

S6 Table Kelderman’s likelihood ratio tests of no DIF for the GLLRM of the PC subscale for Aboriginal Australians. The 5% critical limit for the p-values after adjusting for false discovery rate was p < 0.003. GLLRM: Graphical Loglinear Rasch model. PC: Perceived Constraints Scale.
(DOCX)

S7 Table Kelderman’s likelihood ratio tests no DIF for the RM of the MA subscale for Aboriginal Australians. The 5% critical limit for the p-values after adjusting for false discovery rate was *p* < 0.006.. RM: Rasch model. MA: Mastery Scale.
(DOCX)

S8 Table Kelderman’s likelihood ratio tests no DIF for the GLLRM of the MA subscale for non-Aboriginal Australians. The 5% critical limit for the p-values after adjusting for false discovery rate was *p* < 0.005. GLLRM: Graphical loglinear Rasch model. MA: Mastery Scale.
(DOCX)
