# Supplementary material for: Sense of personal control: Can it be assessed culturally unbiased across Aboriginal and non-Aboriginal Australians?
Source: PLoS One. 2020 Oct 1;15(10):e0239384. doi: 10.1371/journal.pone.0239384 (PMC7529283; doi:10.1371/journal.pone.0239384)
Supplement: S1 Table — (DOCX) [file pone.0239384.s001.docx]

**S1 Table. The Sense of Personal Constrol Scale (SPCS) items compared to items in Pearlin’s Mastery Scale.**

| Item number | Item content | Item present in | | | |
| --- | --- | --- | --- | --- | --- |
|  |  | | Pearlin’s (1981)  Mastery Scale | Lachman and Weaver (1998)  Sense of Personal Control Scale | |
|  |  | |  | Mastery | Perceived Constraints |
| 1 | I can do just about anything I really set my mind to | | X | X |  |
| 2 | Other people decide most of what I can and cannot do | |  |  | X |
| 3 | When I really want to do something I usually find a way to do it | |  | X |  |
| 4 | Whether or not I am able to get what I want is in my own hands | |  | X |  |
| 5 | There is little I can do to change many of the important things in my life | | X |  | X |
| 6 | I often feel helpless in dealing with life’s problems | | X |  | X |
| 7 | There are many things that interfere with what I want to do | |  |  | X |
| 8 | I have little control over the things that happen to me | | X |  | X |
| 9 | There is really no way I can solve all the problems I have | | X |  | X |
| 10 | I sometimes feel I am being pushed around in my life | | X |  | X |
| 11 | What happens to me in the future mostly depends on me | | X | X |  |
| 12 | What happens in my life is often beyond my control | |  |  | X |
