# Supplementary material for: Sense of personal control: Can it be assessed culturally unbiased across Aboriginal and non-Aboriginal Australians?
Source: PLoS One. 2020 Oct 1;15(10):e0239384. doi: 10.1371/journal.pone.0239384 (PMC7529283; doi:10.1371/journal.pone.0239384)
Supplement: S2 Table — Mean values, minimum, maximum and standard deviations; numbers and percentages. TAFE, Technical and Further Education (trade school/college). (DOCX) [file pone.0239384.s002.docx]

**S2 Table. Characteristic of the study participants.**

|  | Teeth Talk Study (Aboriginal Sample) | | National Survey of Adult Oral Health 2004-2006 (Non-Aboriginal Sample) | |
| --- | --- | --- | --- | --- |
|  | n | % | n | % |
| Age |  |  |  |  |
| Mean | 36.4 | | 50.3  14.8  18/82 | |
| SD | 14.0 | |  |  |
| Min/Max | 18/82 | |  |  |
| Missing | 0 | 0% | 0 | 0% |
| Sex |  |  |  |  |
| Female | 214 | 76% | 2388 | 61.9% |
| Male | 103 | 24% | 1469 | 38.1% |
| Missing | 0 | 0% | 0 | 0% |
| Education |  |  |  |  |
| High school or less | 236 | 74% | 1252 | 32.5% |
| TAFE or university | 81 | 26% | 2605 | 67.5% |
| Missing | 0 | 0% | 0 | 0% |
| Employment status |  |  |  |  |
| Job | 80 | 25% | 2947 | 76.4% |
| Benefits | 237 | 75% | 668 | 17.3% |
| Missing | 0 | 0% | 242 | 6.3% |

Note. Mean values, minimum, maximum and standard deviations; numbers and percentages.

TAFE, Technical and Further Education (trade school/college).
