# Supplementary material for: Sense of personal control: Can it be assessed culturally unbiased across Aboriginal and non-Aboriginal Australians?
Source: PLoS One. 2020 Oct 1;15(10):e0239384. doi: 10.1371/journal.pone.0239384 (PMC7529283; doi:10.1371/journal.pone.0239384)
Supplement: S3 Table — CLR: Conditional likelihood ratio. df: degrees of freedom. p: p-value. DIF: differential item function. Overall homogeneity test compares item parameters in approximately equal-sized groups of high and low scoring persons, while the global DIF test for DIF across the entire set of items. The critical limits for the p-values after adjusting for false discovery rate in the GLLRM were: (a)(b) 5% limit p = .05 and 1% limit p = .01. (DOCX) [file pone.0239384.s003.docx]

**S3 Table. Overall fit statistics for the Rasch model for the 12-item SPCS**

| Overall Tests | Aboriginal Australians)^a^ | | |  | Non-Aboriginal Australians)^b^ | | |  |
| --- | --- | --- | --- | --- | --- | --- | --- | --- |
|  | CLR | *df* | *p* |  | CLR | *df* | *p* |  |
| Homogeneity | 273.3 | 47 | <0.001 |  | 1070.7 | 47 | <0.001 |  |
| Global DIF relative to: |  |  |  |  |  |  |  |  |
| Sex | 34.8 | 47 | 0.905 |  | 124.5 | 47 | <0.001 |  |
| Age | 61.0 | 47 | 0.083 |  | 153.8 | 47 | <0.001 |  |
| Education | 78.9 | 47 | 0.002 |  | 380.5 | 47 | <0.001 |  |
| Employment status | 72.0 | 47 | 0.001 |  | 502.1 | 47 | <0.001 |  |

*Notes.* CLR: Conditional likelihood ratio. df: degrees of freedom. p: p-value. DIF: differential item function. Overall homogeneity test compares item parameters in approximately equal-sized groups of high and low scoring persons, while the global DIF test for DIF across the entire set of items. The critical limits for the p-values after adjusting for false discovery rate in the GLLRM were: (a)(b) 5% limit p = .05 and 1% limit p = .01.
