# Supplementary material for: Sense of personal control: Can it be assessed culturally unbiased across Aboriginal and non-Aboriginal Australians?
Source: PLoS One. 2020 Oct 1;15(10):e0239384. doi: 10.1371/journal.pone.0239384 (PMC7529283; doi:10.1371/journal.pone.0239384)
Supplement: S4 Table — PC: Personal Constraints Scale. MA: Mastery Scale. CLR: Conditional likelihood ratio. df: degrees of freedom. p: p-value. DIF: differential item function. Overall homogeneity compares item parameters in approximately equal-sized groups of high and low scoring persons, while the global DIF test for DIF across the entire set of items. The critical limits for the p-values after adjusting for false discovery rate in the GLLRM were: (a) (b) 5% limit p = .05 and 1% limit p = .01; and (c) (d) 5% limit p = .05 and 1% limit p = .01. § The results displayed in this table refer to the original subscales with all items included. (DOCX) [file pone.0239384.s004.docx]

**S4 Table. Overall tests of fit to the Rasch model for the PC and MA subscales^§^.**

| Overall Tests | PC (Aboriginal Australians)^a^ | | |  | MA (Aboriginal Australians)^b^ | | |  | PC (Non-Aboriginal Australians)^c^ | | |  | MA (Non-Aboriginal Australians)^d^ | | |
| --- | --- | --- | --- | --- | --- | --- | --- | --- | --- | --- | --- | --- | --- | --- | --- |
|  | CLR | *df* | *p* |  | CLR | *df* | *p* |  | CLR | *df* | *p* |  | CLR | *df* | *p* |
| Homogeneity | 129.4 | 31 | <0.001 |  | 110.5 | 15 | <0.001 |  | 867.2 | 31 | <0.001 |  | 233.4 | 15 | <0.001 |
| DIF relative to: |  |  |  |  |  |  |  |  |  |  |  |  |  |  |  |
| Sex | 27.7 | 31 | 0.635 |  | 13.6 | 15 | 0.553 |  | 81.5 | 31 | <0.001 |  | 33.3 | 15 | 0.004 |
| Age | 31.1 | 31 | 0.462 |  | 21.7 | 15 | 0.116 |  | 120.5 | 31 | <0.001 |  | 60.4 | 15 | <0.001 |
| Education | 53.2 | 31 | 0.008 |  | 16.2 | 15 | 0.372 |  | 327.2 | 31 | <0.001 |  | 17.5 | 15 | 0.288 |
| Employment status | 48.9 | 31 | 0.021 |  | 18.0 | 15 | 0.262 |  | 443.7 | 31 | <0.001 |  | 36.3 | 15 | 0.002 |

Note*.* PC: Personal Constraints Scale. MA: Mastery Scale. CLR: Conditional likelihood ratio. df: degrees of freedom. p: p-value. DIF: differential item function. Overall homogeneity compares item parameters in approximately equal-sized groups of high and low scoring persons, while the global DIF test for DIF across the entire set of items. The critical limits for the p-values after adjusting for false discovery rate in the GLLRM were: (a) (b) 5% limit p = .05 and 1% limit p = .01; and (c) (d) 5% limit p = .05 and 1% limit p = .01. § The results displayed in this table refer to the original subscales with all items included.
