# Supplementary material for: Sense of personal control: Can it be assessed culturally unbiased across Aboriginal and non-Aboriginal Australians?
Source: PLoS One. 2020 Oct 1;15(10):e0239384. doi: 10.1371/journal.pone.0239384 (PMC7529283; doi:10.1371/journal.pone.0239384)
Supplement: S5 Table — γ = Goodman & Kruskal’s gamma coefficients. PC: Perceived Constraints Scale. MA: Mastery Scale. § The results displayed in this table refer to the original subscales with all items. The critical limits for the p-values after adjusting for false discovery rate in the GLLRM were: (a) 5% limit p = .02 and 1% limit p = .003; (b) 5% limit p = 0.02 and 1% limit p = .004; (c) 5% limit p = 0.04 and 1% limit p = .008; and (d) 5% limit p = 0.04 and 1% limit p = .007. (DOCX) [file pone.0239384.s005.docx]

**S5 Table.** I**tem fit statistics for the Rasch model of the PC and MA subscales.**

|  | Conditional Infit | | | Conditional Outfit | | | Item-restscore association | | |
| --- | --- | --- | --- | --- | --- | --- | --- | --- | --- |
| Items^§^ | Observed | SE | *p* | Observed | SE | *p* | Observed γ | Expected γ | *p* |
| *Aboriginal Australians* | | | | | | | | | |
| PC^a^ |  |  |  |  |  |  |  |  |  |
| 2 | 1.345 | 0.075 | <0.001 | 1.276 | 0.075 | <0.001 | 0.395 | 0.493 | 0.017 |
| 5 | 1.095 | 0.076 | 0.209 | 1.116 | 0.075 | 0.121 | 0.461 | 0.493 | 0.430 |
| 6 | 0.966 | 0.075 | 0.650 | 0.974 | 0.075 | 0.725 | 0.544 | 0.488 | 0.182 |
| 7 | 0.997 | 0.076 | 0.965 | 1.006 | 0.076 | 0.936 | 0.508 | 0.486 | 0.614 |
| 8 | 0.785 | 0.075 | 0.004 | 0.775 | 0.076 | 0.003 | 0.645 | 0.488 | <0.001 |
| 9 | 0.805 | 0.076 | 0.010* | 0.801 | 0.076 | 0.008 | 0.633 | 0.483 | <0.001 |
| 10 | 1.073 | 0.077 | 0.345 | 0.962 | 0.076 | 0.612 | 0.546 | 0.497 | 0.226 |
| 12 | 1.113 | 0.078 | 0.149 | 1.107 | 0.075 | 0.157 | 0.478 | 0.498 | 0.630 |
| MA^b^ |  |  |  |  |  |  |  |  |  |
| 1 | 1.106 | 0.088 | 0.228 | 1.030 | 0.095 | 0.754 | 0.657 | 0.616 | 0.339 |
| 3 | 0.676 | 0.088 | <0.001 | 0.658 | 0.097 | <0.001 | 0.794 | 0.621 | <0.001 |
| 4 | 0.971 | 0.082 | 0.728 | 0.934 | 0.090 | 0.465 | 0.670 | 0.620 | 0.231 |
| 11 | 1.589 | 0.091 | <0.001 | 1.455 | 0.099 | <0.001 | 0.504 | 0.614 | 0.012 |
|  |  |  |  |  |  |  |  |  |  |
| *Non-Aboriginal Australians*  PC^c^ | | | | | | | | | |
| 2 | 1.297 | 0.027 | <0.001 | 1.231 | 0.026 | <0.001 | 0.538 | 0.600 | <0.001 |
| 5 | 1.296 | 0.026 | <0.001 | 1.176 | 0.023 | <0.001 | 0.569 | 0.617 | <0.001 |
| 6 | 0.876 | 0.026 | <0.001 | 0.853 | 0.025 | <0.001 | 0.709 | 0.607 | <0.001 |
| 7 | 1.249 | 0.024 | <0.001 | 1.130 | 0.022 | <0.001 | 0.560 | 0.601 | <0.001 |
| 8 | 0.765 | 0.027 | <0.001 | 0.761 | 0.025 | <0.001 | 0.747 | 0.606 | <0.001 |
| 9 | 0.876 | 0.027 | <0.001 | 0.884 | 0.025 | <0.001 | 0.690 | 0.612 | <0.001 |
| 10 | 1.003 | 0.025 | 0.914 | 0.987 | 0.023 | 0.590 | 0.639 | 0.609 | 0.006 |
| 12 | 1.075 | 0.025 | 0.003 | 1.021 | 0.023 | 0.367 | 0.631 | 0.608 | 0.041 |
|  |  |  |  |  |  |  |  |  |  |
| MA^d^ |  |  |  |  |  |  |  |  |  |
| 1 | 0.926 | 0.032 | 0.022 | 1.000 | 0.027 | 0.997 | 0.701 | 0.711 | 0.490 |
| 3 | 0.804 | 0.036 | <0.001 | 0.882 | 0.029 | <0.001 | 0.786 | 0.754 | 0.021 |
| 4 | 0.873 | 0.032 | <0.001 | 0.903 | 0.025 | <0.001 | 0.596 | 0.579 | 0.334 |
| 11 | 1.303 | 0.035 | <0.001 | 1.240 | 0.029 | <0.001 | 0.596 | 0.579 | 0.334 |

Note. γ = Goodman & Kruskal’s gamma coefficients. PC: Perceived Constraints Scale. MA: Mastery Scale. § The results displayed in this table refer to the original subscales with all items. The critical limits for the p-values after adjusting for false discovery rate in the GLLRM were: (a) 5% limit p = .02 and 1% limit p = .003; (b) 5% limit p = 0.02 and 1% limit p = .004; (c) 5% limit p = 0.04 and 1% limit p = .008; and (d) 5% limit p = 0.04 and 1% limit p = .007.
