# Supplementary material for: Sense of personal control: Can it be assessed culturally unbiased across Aboriginal and non-Aboriginal Australians?
Source: PLoS One. 2020 Oct 1;15(10):e0239384. doi: 10.1371/journal.pone.0239384 (PMC7529283; doi:10.1371/journal.pone.0239384)
Supplement: S6 Table — The 5% critical limit for the p-values after adjusting for false discovery rate was p < 0.003. GLLRM: Graphical Loglinear Rasch model. PC: Perceived Constraints Scale. (DOCX) [file pone.0239384.s006.docx]

**S6 Table. Kelderman’s likelihood ratio tests of no DIF for the GLLRM of the PC subscale for Aboriginal Australians.**

|  | Conditional Likelihood Ratio test | | |
| --- | --- | --- | --- |
| Item 2 & Sex: | lr =    0.21 | df =   4 | p = 0.99 |
| Item 5 & Sex: | lr =   10.07 | df =   4 | p = 0.04 |
| Item 7 & Sex: | lr =    2.35 | df =   4 | p = 0.67 |
| Item 10 & Sex: | lr =    4.00 | df =   4 | p = 0.41 |
| Item 12 & Sex: | lr =    3.05 | df =   4 | p = 0.55 |
| Item 2 & Education: | lr =    8.27 | df =   4 | p = 0.08 |
| Item 5 & Education: | lr =    3.27 | df =   4 | p = 0.51 |
| Item 7 & Education: | lr =   10.21 | df =   4 | p = 0.04 |
| Item 10 & Education: | lr =    4.25 | df =   4 | p = 0.37 |
| Item 12 & Education: | lr =    0.66 | df =   4 | p = 0.95 |
| Item 2 & Employment status: | lr =    5.40 | df =   4 | p = 0.25 |
| Item 5 & Employment status: | lr =    4.77 | df =   4 | p = 0.31 |
| Item 7 & Employment status: | lr =    4.28 | df =   4 | p = 0.37 |
| Item 10 & Employment status: | lr =    1.58 | df =   4 | p = 0.81 |
| Item 12 & Employment status: | lr =    9.54 | df =   4 | p = 0.05 |
| Item 2 & Age: | lr =    2.92 | df =   4 | p = 0.57 |
| Item 5 & Age: | lr =    5.21 | df =   4 | p = 0.27 |
| Item 7 & Age: | lr =    4.58 | df =   4 | p = 0.33 |
| Item 10 & Age: | lr =    4.05 | df =   4 | p = 0.40 |
| Item 12 & Age: | lr =    9.26 | df =   4 | p = 0.05 |

Note. The 5% critical limit for the p-values after adjusting for false discovery rate was *p* < 0.003. GLLRM: Graphical Loglinear Rasch model. PC: Perceived Constraints Scale
