# Supplementary material for: Sense of personal control: Can it be assessed culturally unbiased across Aboriginal and non-Aboriginal Australians?
Source: PLoS One. 2020 Oct 1;15(10):e0239384. doi: 10.1371/journal.pone.0239384 (PMC7529283; doi:10.1371/journal.pone.0239384)
Supplement: S7 Table — The 5% critical limit for the p-values after adjusting for false discovery rate was p < 0.006.. RM: Rasch model. MA: Mastery Scale. (DOCX) [file pone.0239384.s007.docx]

**S7 Table. Kelderman’s likelihood ratio tests no DIF for the RM of the MA subscale for Aboriginal Australians.**

|  | Conditional Likelihood Ratio test | | |
| --- | --- | --- | --- |
| Item 1 & Sex: | lr =    4.39 | df =   4 | p = 0.36 |
| Item 3 & Sex: | lr =    0.31 | df =   4 | p = 0.96 |
| Item 1 & Education: | lr =    4.41 | df =   4 | p = 0.35 |
| Item 3 & Education: | lr =    3.97 | df =   4 | p = 0.27 |
| Item 1 & Employment status: | lr =    9.81 | df =   4 | p = 0.04 |
| Item 3 & Employment status: | lr =    5.47 | df =   4 | p = 0.14 |
| Item 1 & Age: | lr =    3.36 | df =   4 | p = 0.50 |
| Item 3 & Age: | lr =    5.21 | df =   4 | p = 0.16 |

Note. The 5% critical limit for the p-values after adjusting for false discovery rate was *p* < 0.006.. RM: Rasch model. MA: Mastery Scale.
