# Supplementary material for: Sense of personal control: Can it be assessed culturally unbiased across Aboriginal and non-Aboriginal Australians?
Source: PLoS One. 2020 Oct 1;15(10):e0239384. doi: 10.1371/journal.pone.0239384 (PMC7529283; doi:10.1371/journal.pone.0239384)
Supplement: S8 Table — The 5% critical limit for the p-values after adjusting for false discovery rate was p < 0.005. GLLRM: Graphical loglinear Rasch model. MA: Mastery Scale. (DOCX) [file pone.0239384.s008.docx]

**S8 Table. Kelderman’s likelihood ratio tests no DIF for the GLLRM of the MA subscale for Non-Aboriginal Australians.**

|  | Conditional Likelihood Ratio test | | |
| --- | --- | --- | --- |
| Item 3 & Sex: | lr =    4.90 | df =   4 | p = 0.30 |
| Item 4 & Sex: | lr =    6.20 | df =   4 | p = 0.18 |
| Item 1 & Education: | lr =    3.02 | df =   4 | p = 0.55 |
| Item 3 & Education: | lr =    5.99 | df =   4 | p = 0.20 |
| Item 4 & Education: | lr =    2.55 | df =   4 | p = 0.63 |
| Item 1 & Employment status: | lr =    3.24 | df =   4 | p = 0.52 |
| Item 3 & Employment status: | lr =    8.32 | df =   4 | p = 0.08 |
| Item 4 & Employment status: | lr =    5.40 | df =   4 | p = 0.25 |
| Item 3 & Age: | lr =    15.24 | df =   4 | p = 0.004 |
| Item 4 & Age: | lr =    5.31 | df =   4 | p = 0.25 |

Note. The 5% critical limit for the p-values after adjusting for false discovery rate was *p* < 0.005. GLLRM: Graphical loglinear Rasch model. MA: Mastery Scale.
